# Spatial relationship between land-use/land-cover change and land surface temperature in the Dongting Lake area, China

**DOI:** 10.1038/s41598-020-66168-6

**Published:** 2020-06-08

**Authors:** Jie Tan, De Yu, Qiang Li, Xuelan Tan, Weijun Zhou

**Affiliations:** 1 College of Landscape Architecture and Art Design, Hunan Agricultural University, Changsha, 410128 China; 2 School of Public Administration, China University of Geosciences, Wuhan, 430074 China; 3 Hunan Hydro & Power Design Institute, Changsha, 410007 China; 4 College of Resources and Environment, Hunan Agricultural University, Changsha, 410128 China

**Keywords:** Attribution, Wetlands ecology

## Abstract

The Dongting Lake area (China) is a climate change-sensitive and ecologically fragile area and plays a crucial role in the regulation of the regional climate. In recent decades, rapid social and economic development has led to increased land use/land cover (LULC) changes in the Dongting Lake area, which affect the surface energy balance and hydrological processes. Its contemporary variability under climate change remains highly uncertain. Therefore, we retrieved the Land surface temperature (LST) from the Landsat 7 data and explored its relationship with the LULC types. The results showed that LST is significantly affected by surface type. LST varied significantly across LULC types, with higher LSTs in built-up land, reed beach land, forest land, and paddy fields than in water bodies, mud beaches, marshlands, and riparian forests. Water bodies play an important regulatory role in reducing LST and mitigating thermal effects on the ground. The winter LST in the study area increased by approximately 3.5 °C, which may be related to the decrease in the area of Dongting Lake water bodies, water fields and reed flats after the Three Gorges Reservoir was impounded. Compared with the relationship between the NDVI, DEM, and distance from the water body, the negative correlation between the NDMI and LST was stronger and more stable and had the greatest effect on LST. These insights improve the understanding of the land change consequences on the temporal dynamics of LST.

## Introduction

Land surface temperature (LST) is a reflection of the energy flow in the interactions between the land surface and atmosphere and between the land surface and biosphere. The change in LST is an intuitive regional climate response to global climate change and has important research significance in agriculture, hydrology, ecology, environment, climate, and biogeochemistry1^1–6^. LULC is the interface between the atmosphere and biosphere for material and energy exchange and has a clear influence on LST. Currently, a wide consensus has been reached on the influence of LULC change on regional climate through various processes, such as water and heat flux between land and air^7^, surface wind speed, and boundary layer turbulence8^8,9^. Exploring the mechanism of interaction between LULC change and local climate has become a popular research topic^10–12^. LULC change directly causes changes in the physical characteristics of the land surface, which affects a variety of factors that determine regional climate, such as radiation, heat, and water vapor exchange^13–15^. LULC change also alters the composition of surface attachments such as vegetation type and vegetation density^16,17^. In addition, LULC change has associated effects such as demographic changes and changes in regional chemical composition^18,19^. This relationship has led to changes in terrestrial carbon stocks and their fluxes^20–22^, resulting in a variety of outcomes, including changes in atmospheric greenhouse gas levels^23–25^. Therefore, the relationship between LST and LUC change should be investigated to further address regional environmental problems and provide a basis for regional planning.

Although the use of meteorological data for regional climate change research has progressed, meteorological data are difficult to obtain, and data accuracy is dependent on the site and the scope of the investigation, resulting in difficulties in achieving large-scale and long-term research. Compared with traditional observation methods used at meteorological stations, monitoring LST with remote sensing offers the advantages of a wide observation range and strong spatial continuity. Thus, this monitoring has developed rapidly in thermal environment research^26–28^. To achieve surface temperature inversion of satellite thermal infrared data, it is necessary to address the effects of atmospheric interference and surface incidence. Researchers have proposed different ways to eliminate these disturbances. The current methods for LST inversion using Landsat/TM 6-band data are mainly single-window algorithms^29^, single-channel algorithms^30^ and atmospheric correction methods based on radiation transmission equations^31^. The spatial distribution trend of LST obtained by the three inverse algorithms is consistent with the measured surface temperature, the results of the radiation transmission equation algorithm are slightly higher than the measured surface value, the results of the single-window algorithm are the most consistent with the measured surface value, and the results of the single-channel algorithm are significantly lower than the measured surface value.

The Dongting Lake area is a typical water-land eco-fragile zone that has been strongly affected by human activities in recent decades. In particular, the Three Gorges Dam began to impound water on June 1, 2003, which accelerated the shrinking of water bodies and significantly altered the land cover state in the Dongting Lake area, and this process has been accompanied by many discussions about its environmental impacts. Some studies have said the dam has had important impacts on the elements of the regional climate system and the interactions between the land surface and the atmosphere. Specifically, it has changed the distribution pattern of regional precipitation and temperature. To verify the impact of the Three Gorges Dam on environmental changes, especially the relationship between LULC change and LST, we used U.S. Geological Survey Landsat TM/ETM + data to obtain LULC change and winter LST information for the Dongting Lake area in this study. Thus, we could better understand the influence of human activities on regional LST and its response to global warming. The exploration of such regional LULC and climate change patterns and processes can provide a typical case for a larger range of global change studies.

## Extraction of LULC change information and inversion of LST

### Research areas and data

**Figure 1:**
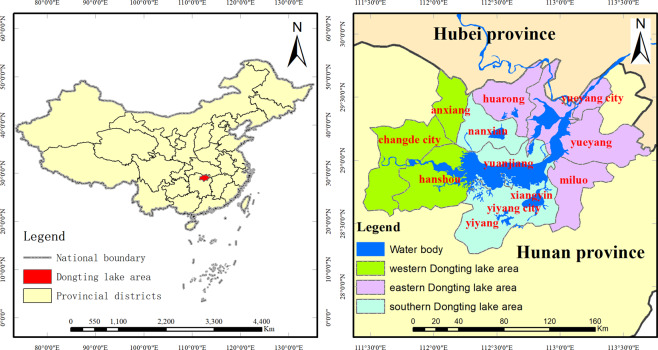
Location of the study area.

The Dongting Lake area is located in the middle and lower reaches of the Yangtze River (28°30′N–30°20′N, 111°40′E–113°10′E), mostly below 50 m above sea level (Fig. 1). It is a typical lake basin in the middle and low latitudes, with a mean annual temperature of 16.5–17.2 °C and an annual precipitation of 1289.8–1556.2 mm. The Dongting Lake area has an East Asian monsoon climate, and the climate zone transitions from the mid-subtropical zone to the north subtropical zone. As China’s largest ecological landscape system of freshwater lake wetlands, its biological resources are rich. The Dongting Lake area is in the transitional zone of different ecological landscapes of the Yangtze River basin^32^, occupying the most sensitive and vulnerable ecological location in the middle and lower reaches of the Yangtze River. This area is important in terms of regulating temperature and humidity and maintaining regional climate stability, biodiversity conservation, and water purification. Studies have shown that from 1952 to 2010, the temperature in the Dongting Lake area increased significantly, the alternation of the cold and warm period was more frequent, and the frequency of extreme precipitation and heavy rain increased considerably^33,34^.

To analyse the change in land use/land cover and its impact on LST in the Donting Lake area before and after Three Gorges impoundment of the Yangtze River, we selected 16 remote sensing images from the winters of 1995, 2001, 2004 and 2013 as the main data sources (Table 1). It should be noted that we did not use the 1993 and 1994 data because there are no suitable data available. We obtained LULC change data using visible light bands (spatial resolution of 30 m, with cloudiness of 0%) and obtained the LST using the thermal infrared band (spatial resolution of 120 m or 60 m, with cloudiness of 0%). Based on the digital elevation model (DEM) data of 30 m spatial resolution, the administrative division data for the second land survey period, the Moderate Resolution Imaging Spectroradiometer (MODIS) temperature products and meteorological data, and the 2004 ENVI® software (Harris Geospatial Solutions, Broomfield, CO) data, we conducted pre-treatment tasks of radiation calibration, atmospheric correction, geometric correction, cutting, and multisource dataset establishment on the 1995, 2001, and 2013 images. The images of 1995, 2001, and 2004 are TM images, with a spatial resolution of 120 m for band 6, while the spatial resolution of the 2013 ETM + data for band 6 is 60 m. For better comparison of the annual LST data, we resampled the spatial resolution of each year to 120 m.

**Table 1:** Information on remote sensing images.

Path/Row	images of 1995	images of 2001	images of 2004	images of 2013
123/39	1995–12–05	2002–01–05	2004–12–13	2014–01–15
123/40	1995–12–05	2002–01–05	2004–12–13	2013–12–30
124/39	1995–12–28	2001–12–29	2004–11–18	2014–01–22
124/40	1995–12–28	2001–12–29	2004–11–18	2014–01–22

### Extraction of LULC information

**Figure 2:**
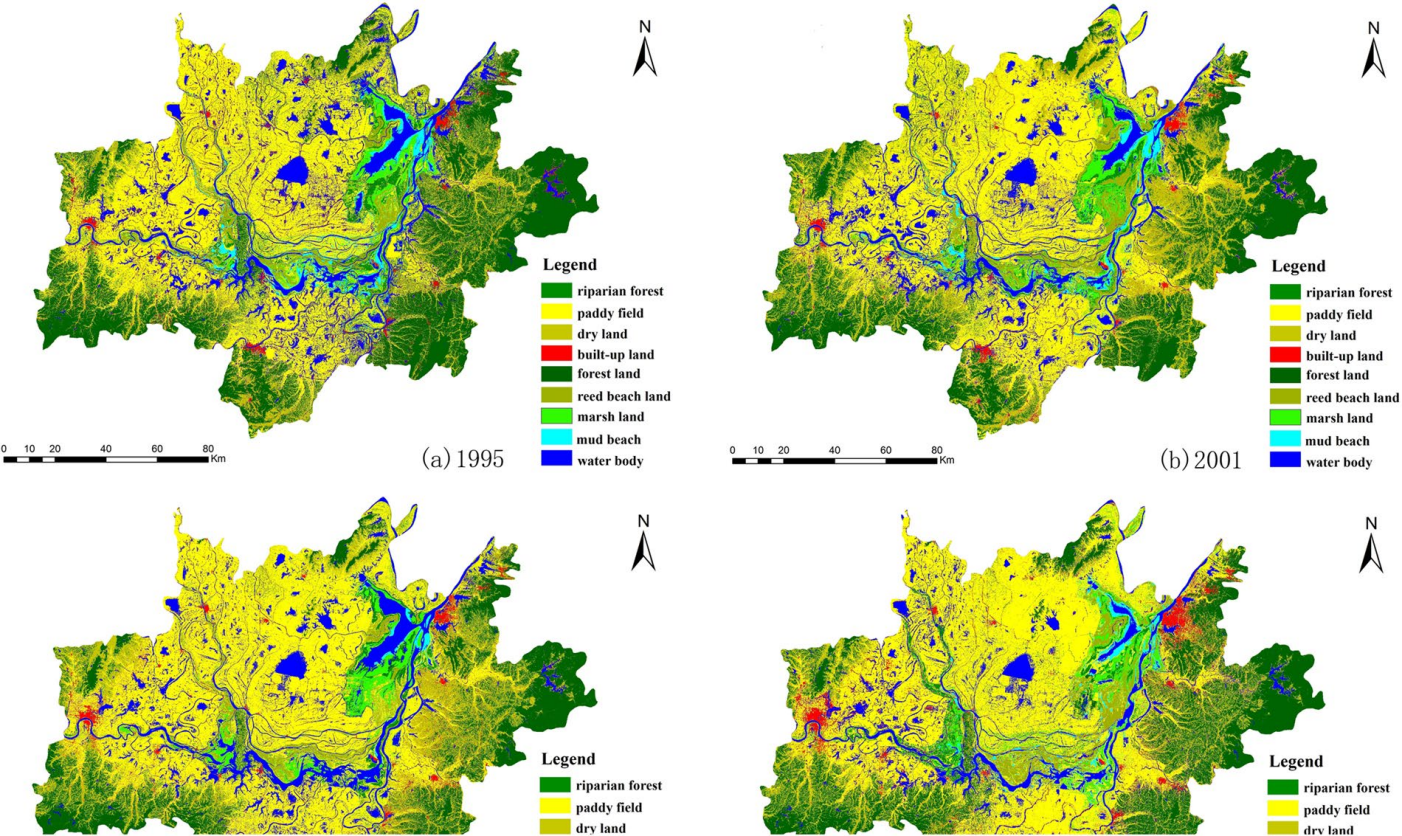
LULC classification maps in Dongting Lake area in 1995, 2001, 2004 and 2013.

To reduce the classification error caused by the large study area, we first divided the study area into three parts, namely, the eastern Dongting Lake area, the southern Dongting Lake area, and the western Dongting Lake area. Based on the the Convention on Wetlands of International Importance especially as Waterfowl Habitat and actual situation of the study area and the characteristics of the remote-sensing image, we divided the Dongting Lake area’s LULC types into nine categories—water body, mud beach, marshland, reed beach land, riparian forest, paddy field, dryland, built-up land, and forest land. According to the high-resolution Google Earth images and field survey, we identified training samples to classified the LULC using the decision tree methodology which is based on the classification and regression tree (CART) algorithm^35–37^, and selected more than 800 pixels in each land type as ground truth region of interest (GTROIs) to verify classification accuracy using the confusion matrix methodology in the ENVI® software. Then we obtained the LULC classification maps in the Dongting Lake area (Fig. 2). The results of the precision validation showed that the overall classification accuracy was more than 85% in 1995, 2002, 2004, and 2013, and the kappa coefficient was greater than 0.8, which met the requirement of a large area LULC change analysis (Table 2).

**Table 2:** The accuracy of classified LULC.

Year	Region	Overall accuracy (%)	Kappa coefficient
1995	Western Dongting Lake area	97.72	0.97
	Southern Dongting Lake area	96.82	0.96
	Eastern Dongting Lake area	89.81	0.88
2001	Western Dongting Lake area	93.21	0.92
	Southern Dongting Lake area	86.75	0.86
	Eastern Dongting Lake area	90.34	0.88
2004	Western Dongting Lake area	93.47	0.93
	Southern Dongting Lake area	94.96	0.95
	Eastern Dongting Lake area	97.52	0.97
2013	Western Dongting Lake area	88.75	0.87
	Southern Dongting Lake area	87.34	0.85
	Eastern Dongting Lake area	90.82	0.89

### LST retrieval and grading

The single-window algorithm is derived by Qin *et al. 29* based on the surface thermal radiation conduction equation, which uses Landsat TM/ETM + data to invert LST. The single-window algorithm has the greatest consistency with the actual measurements. We compared the retrieval results of 2001, 2004, and 2013 with the MODIS temperature products from the same time, and the results indicated a relative error of 2% or less.

1 $$LST=\frac{1}{C}[a(1-C-D)+(b(1-C-D)+C+D){T}_{6}-D{T}_{a}]$$

where C = *ε*_6_ × *τ*_6_ (*ε*_6_ is the surface emissivity; *τ*_6_ is the atmospheric transmittance), *D* = (1 − *τ*_6_)[1 + *τ*_6_(1 − *ε*_6_)], *T*_6_ is the land surface brightness temperature, and *T*_*a*_is the mean atmospheric temperature. The values of *a* in the TM and ETM+ sensors are −67.35535 and −63.18853, respectively, and the values of *b* are −0.458608 and 0.44411, respectively.

The acquisition time for each image was in the winter. To reduce the influence of other factors, such as rainfall or snowfall, on the LST retrieval results and increase the comparability of the results, we standardized the results of the retrieval using a normalization method. The LST distribution range was standardized between 0 and 1, and the normalization formula is as follows:

2 $${N}_{i}=\frac{LS{T}_{i}-LS{T}_{min}}{LS{T}_{max}-LS{T}_{min}}$$

where *N*_*i*_ is the normalized pixel value; *LST*_*i*_ is the retrieval of LST for pixel *i*; *LST*_*max*_ is the maximum LST value in the range; and *LST*_*min*_ is the minimum LST value in the range.

We graded LST using standard deviation classification and obtained the annual temperature grading of the Dongting Lake area (Table 3).

**Table 3:** Standard deviation classification.

Grade	Grade scale	1995	2001	2004	2013
Very low temperature region	<m - 2.5 s	0.0000~0.2450	0.0000~0.2589	0.0000~0.2360	0.0000~0.2599
Low temperature region	m - 2.5 s~m - 1.5 s	0.2450~0.3250	0.2589~0.2988	0.2360~0.3102	0.2599~0.3738
Relatively low temperature region	m - 1.5 s~m - 0.5 s	0.3250~0.4050	0.2988~0.3387	0.3102~0.3844	0.3738~0.4878
Intermediate temperature region	m ± 0.5 s	0.4050~0.4849	0.2988~0.3387	0.3844~0.4586	0.4878~0.6018
Relatively high temperature region	m + 0.5 s~m + 1.5 s	0.4849~0.5649	0.3786~0.4185	0.4586~0.5328	0.6018~0.7158
High temperature region	m + 1.5 s~m + 2.5 s	0.5649~0.6449	0.4185~0.4584	0.5328~0.6070	0.7158~0.8298
Very high temperature region	>m + 2.5 s	0.6449~1.0000	0.4584~1.0000	0.6070~1.0000	0.8298~1.0000

### Statistical analysis

Using overlay analysis in ArcGIS software (Esri Redlands, CA), we calculated the mean temperature of each LULC type and the change in the area of each temperature grade. We graded the DEM, NDVI, NDMI and distance from the water body at regular intervals (NDVI and NDMI were in steps of 0.01 m, digital elevation was in steps of 10 m and distance from water body was in steps of 100 m). Then, we used correlation analysis to reveal the relationships between LST and these factors.

3 $${m}_{t}=\frac{{\sum }_{i=1}^{n}LS{T}_{i}}{n}{m}_{t,j}=\frac{{\sum }_{k=1}^{m}LS{T}_{j,k}}{m}(j=1,2,3\ldots \ldots 9){D}_{t,j}={m}_{t,j}-{m}_{t}$$

where *m*_*t*_ is the mean LST value of the whole study area in year *t*; *LST*_*i*_ is the LST value of pixel *i*; *m*_*t,j*_ is the mean LST value of land type *j* in year *t*; *LST*_*j,k*_ is the LST value of pixel *k* in land type *j*; *D*_*t,j*_ is the difference between the mean LST value of land type *j* and the LST value of the whole study area in year *t*.

## Results

### The dynamics of LULC change

**Figure 3:**
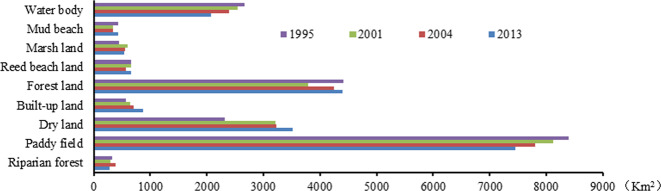
Changes in LULC in the Dongting Lake area in 1995, 2001, 2004 and 2013.

In Fig. 3, the Dongting Lake LULC types were composed mainly of paddy fields, forest lands, drylands, and water bodies. The total area of these four LULC types accounted for 88.06%, 87.38%, 87.38%, and 86.29%, respectively, of the total area in 2013. Between 1995 and 2013, the paddy field and water body areas decreased continuously. The paddy field decreased from 8399.32 km^2^ to 7449.11 km^2^, and the water body decreased from 2670.59 km^2^ to 2078.12 km^2^, a reduction of 11.31% and 22.18%, respectively. Dryland and built-up land areas increased continuously. The dryland area increased by 52.23% from 1995 to 2013, and its proportion in the total land area increased from 11.44% to 17.42%. The built-up land area in 2013 increased by 54.73% compared with that in 1995. Its proportion in the total area increased from 2.79% to 4.32%. Changes in other LULC types were relatively small. Affected by the policy of returning crops to lakes and the successful construction of the Three Gorges Dam, which began to impound water in 2003, the river’s water shortage in the middle and lower reaches of the Yangtze River has led to an accelerated decline in the area of water bodies and paddy fields.

### LST for different LULC types

The mean LST of the Dongting Lake area in the last 20 years increased from 7.13 °C to 11.01 °C, an increase of approximately 3.5 °C, which is consistent with the global warming trend (Table 4). The mean LST of dryland, built-up land, reed beach land, forest land, and paddy field were generally higher than the LST of water body, mud beach, marshland, and riparian forest. From the differences between the mean temperature of various LULC types and the mean temperature of the Dongting Lake area, it is evident that the mean temperature of riparian forest, marshland, mud beach and water body were lower than the mean temperature, and the temperature difference was the largest for the water body, which was the LULC type that had the lowest temperature in winter. From differences between the mean temperature of the same LULC types and the mean temperature of the Dongting Lake area, it is evident that the difference between the mean temperature of each LULC type and the mean temperature of Dongting Lake decreased over time, which indicates that the spatial temperature difference of Dongting Lake is decreasing. In Table 4, *m*_1995_, *m*_2001_, *m*_2004_ and *m*_2013_ represent the mean land surface temperature in each year. D1, D2, D3, and D4 represent the difference between the average temperature of the land type and the average temperature of the Dongting Lake area in 1995, 2001, 2004 and 2013, respectively.

**Table 4:** The mean LST statistics of LULC types (°C).

LULC types	*m*_1995_ (°C)	*m*_2001_ (°C)	*m*_2004_ (°C)	*m*_2013_ (°C)	D1(°C)	D2(°C)	D3(°C)	D4(°C)
Riparian forest	6.61	9.39	9.08	11.10	−0.52	−0.45	−0.69	0.09
Paddy field	7.64	9.91	10.21	11.07	0.51	0.07	0.44	0.06
Dryland	8.08	10.50	10.34	11.16	0.95	0.66	0.57	0.15
Built-up land	8.07	10.44	10.32	11.02	0.94	0.60	0.55	0.01
Forest land	7.75	10.50	10.48	11.27	0.62	0.66	0.71	0.26
Reed beach land	7.79	10.91	10.24	11.47	0.66	1.07	0.47	0.46
Marshland	6.89	10.29	9.12	10.96	−0.24	0.45	−0.65	−0.05
Mud beach	5.51	9.18	9.34	10.90	−1.62	−0.66	−0.43	−0.11
Water body	5.82	7.43	8.79	10.18	−1.31	−2.41	−0.98	−0.83
Total	7.13	9.84	9.77	11.01	—	—	—	—

### Temporal and spatial variability of the LST

**Figure 4:**
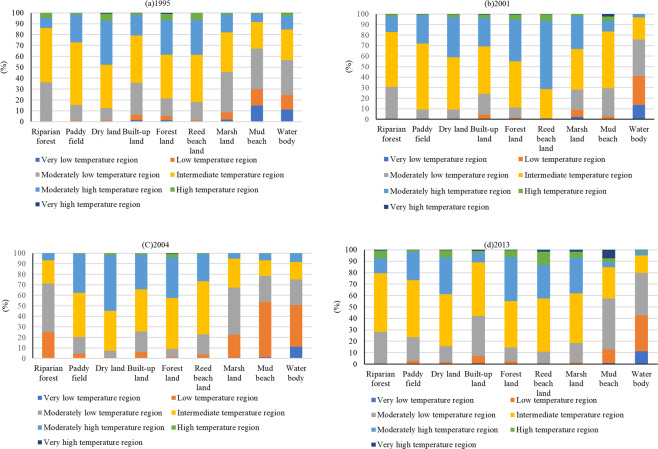
The LST grade area proportion of landscape types in 1995, 2001, 2004, and 2013.

In Fig. 4, the Dongting Lake area mainly had medium-, moderately high-, and moderately low-temperature regions in winter. The areas of low- and extremely low-temperature regions were larger than those of high- and extremely high-temperature regions. There were significant differences in the distribution of LST-graded regions for different LULC types. Water bodies accounted for 56.55%, 75.65%, 75.06%, and 79.67% of the extremely low-, low-, and moderately low-temperature regions in 1995, 2001, 2004, and 2013, respectively, showing a significant increasing trend. The water body has an essential regulatory role in reducing LST and alleviating the thermal effect of the ground. Muddy beach and marshland also occupied a large proportion of the extremely low-, low-, and moderately low-temperature regions. From 1995 to 2013, the proportion of marshland was 45.79%, 27.99%, 67.39%, and 18.52%, respectively. The proportions of muddy beach were 67.08%, 29.32%, 78.14%, and 57.29%, respectively. The proportions of riparian forest were 36.28%, 30.77%, 71.25%, and 28.33%, respectively. These results indicate that marshland, mud beach, and riparian forest are also the main regions of low temperature. The proportions of extremely high-, high-, and moderately high-temperature regions in forest land and dryland were always above 38%, which indicated that in winter, the temperature of forest land and dryland was higher than that of other lands. Paddy field mainly contained medium-temperature regions, which had a proportion of approximately 50% across all years. The proportion of high-temperature regions was maintained at approximately 30%. The temperature distribution in built-up land was relatively balanced, which mainly consisted of medium-temperature regions. The proportion of medium-temperature regions was between 40% and 50% across the years. The areas of low-temperature and high-temperature regions were similar.

### Correlation between LST and other factors (NDVI, NDMI, DEM and distance from water body)

**Figure 5:**
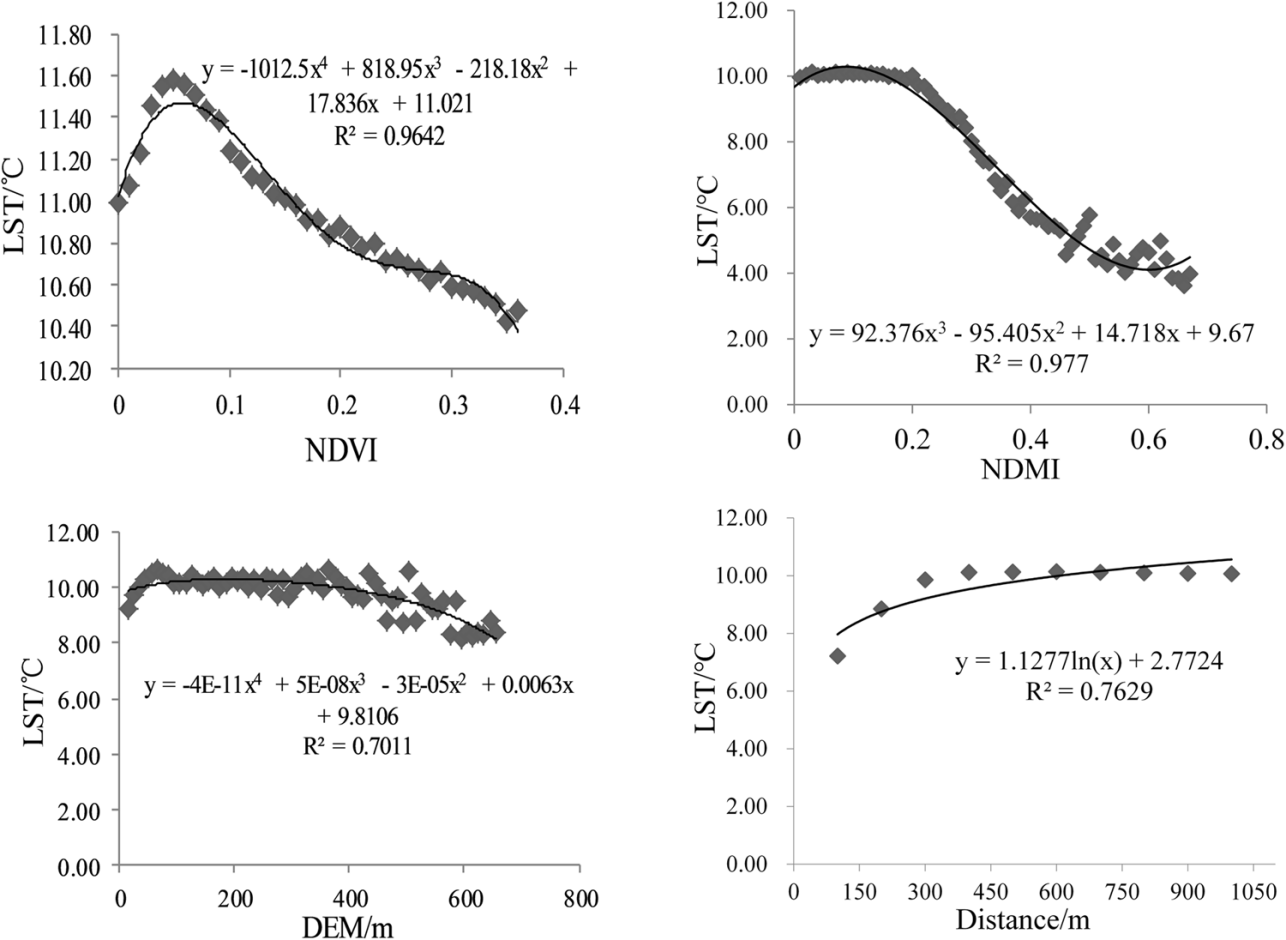
Correlation between LST and NDVI, NDMI, DEM, and distance from water body.

In terms of the correlation of mean LST with these factors (Fig. 5), the NDMI had the greatest effect on LST change, followed by distance, NDVI, and DEM. The effect of the NDMI was significant and negatively correlated with the mean LST. With an increase in the NDMI, the mean LST decreased significantly, with a range of up to 6 °C. When the NDMI was less than 0.2, the mean temperature change was not evident. When the NDMI was greater than 0.2, the mean LST decreased significantly. The trend of temperature decline weakened when the NDMI decreased to approximately 0.5, showing that water vapor had a significant cooling effect on LST. The NDVI was positively correlated with mean LST when NDVI was between 0 and 0.05 and was negatively correlated with mean LST when NDVI was greater than 0.05. Although the NDVI had a linear relationship with the mean LST, the change in the mean LST, which was less than 2 °C, was not obvious with the NDVI. Therefore, the effect of NDVI on mean LST was minimal in winter, which may have been because winter is not a growth period for vegetation. When the DEM was <65 m, the LST was positively correlated with the DEM. In this range, the LULC types in the Dongting Lake area were mainly water body, beach land, and paddy field, and the LST was affected by the cooling effect of the water body, the underlying surface moisture, and the surface coverage. When the DEM was between 65 m and 365 m, the surface cover did not change, and the mean LST was relatively stable. When the DEM was greater than 365 m, the DEM had a weak negative correlation with the mean LST. The LST was significantly positively correlated with distance when the distance from the water body was less than 300 m. When the distance exceeds 300 m, there was no significant correlation between them. This indicated that the water body has a certain cooling effect on the thermal environment within 300 meters of its surroundings, and the ecological protection of the water body and spatial layout planning are important to mitigate the urban heat-island effect.

## Discussion

Observational analyses show that the surface temperature changes in China in recent decades have shown significant warming in the north but not in the south, especially in the southwest and parts of the lower Yangtze River^38,39^. Northern and southwestern China are more sensitive to climate change^40^, while areas south of the Yellow River are less sensitive. We demonstrate that China’s urbanization has resulted in the expansion of construction land in the Dongting Lake area, i.e., the implementation of the Three Gorges Dam water storage, and the “return of land to forest” policy, which has resulted in the shrinking of water bodies and rice fields and the growth of drylands. These factors work together to cause the average LST to show an increasing trend over the winter. The impacts of land use/cover types and land cover changes are more regional than the global impacts of increased GHGs. This result has also been confirmed in many regional climate change studies^41,42^. The results of the correlation analysis also show that vegetation cover, air humidity, and distance from water bodies can significantly regulate surface temperature, which is similar to the findings of many studies on urban thermal environments^43–46^. Therefore, managers need to pay more attention to the protection of the ecological functions of wetlands and rice paddies and implement ecological restoration projects under the concept of the community of life of mountains, water, fields, lakes, grass and people.

Some studies have shown that the LST algorithm has excluded atmospheric radiation information, and the influence of atmospheric composition on LST no longer needs to be considered when analysing factors affecting LST^47^. However, the LST difference is affected not only by the difference in solar radiation but also by the surface cover, human activities, precipitation, air flow, and landscape pattern. Future research must consider factors such as airflow, monsoons, rainfall, solar radiation, seasonal climate change, and landscape patterns^48–52^. The quantitative relationship between each factor and temperature needs to be established, and the mathematical model needs to simulate climate change. More thorough and detailed studies are needed to understand the dynamic mechanisms of climate change in the Dongting Lake area and to further assess the impact of human activities on regional climate change.

## Conclusion

In this study, LST was extracted from Landsat TM/ETM+ data using a single-window algorithm, the differences in LST among different LULC types were analysed, the spatial distribution characteristics of LST and the NDMI, NDVI, DEM, and distance were discussed, their quantitative relationships were determined, and the spatiotemporal distribution laws and influencing factors of LST were clarified. The main conclusions were as follows. (1) The LULC type in the Dongting Lake area has changed significantly, with the area of water body and paddy field declining continuously and the area of built-up land and dryland increasing gradually; these results indicate that the changes in the LULC type in the area are related to the water impoundment of the Three Gorges Dam and the ecological management policies. (2) LST in the Dongting Lake area increased by 3.5 °C in winter, with differences in LST of different LULC types, LST of forest land and dryland is higher than that of other land types, and the LST of water body is much lower than that of other land types, also is much lower than the mean LST of the study area. In addition, within 300 m to the water body, the closer to the water body, the lower the LST. It suggests that the water body has a moderating effect on the LST and surface thermal effects at a certain distance around it. (3) The NDMI showed a strong negative spatial correlation with LST, while the NDVI, DEM and distance showed a weak spatial correlation with LST. In winter, water vapor plays a major role in altering the pattern of heat distribution on the lower mat, and the NDMI is a valid indicator for thermal environment studies and is more suitable for quantitative analysis of LST. Digital remote sensing methods can not only provide a range of surface temperatures across a region but also demonstrate its spatial pattern. These results have increased the understanding of the consequences of land change on the spatiotemporal dynamics of LST.
